# Specificity of assemblage, not fungal partner species, explains mycorrhizal partnerships of mycoheterotrophic *Burmannia* plants

**DOI:** 10.1038/s41396-020-00874-x

**Published:** 2021-01-06

**Authors:** Zhongtao Zhao, Xiaojuan Li, Ming Fai Liu, Vincent S. F. T. Merckx, Richard M. K. Saunders, Dianxiang Zhang

**Affiliations:** 1 Key Laboratory of Plant Resources Conservation and Sustainable Utilization, South China Botanical Garden, Chinese Academy of Sciences, Guangzhou, 510650 China; 2 Division of Ecology & Biodiversity, School of Biological Sciences, The University of Hong Kong, Pokfulam Road, Hong Kong, China; 3 Naturalis Biodiversity Center, 2332 AA Leiden, The Netherlands; 4 Department of Evolutionary and Population Biology, Institute for Biodiversity and Ecosystem Dynamics, University of Amsterdam, Amsterdam, The Netherlands

**Keywords:** Fungi, Plant sciences, Evolution

## Abstract

Mycoheterotrophic plants (MHPs) growing on arbuscular mycorrhizal fungi (AMF) usually maintain specialized mycorrhizal associations. The level of specificity varies between MHPs, although it remains largely unknown whether interactions with mycorrhizal fungi differ by plant lineage, species, and/or by population. Here, we investigate the mycorrhizal interactions among *Burmannia* species (Burmanniaceae) with different trophic modes using high-throughput DNA sequencing. We characterized the inter- and intraspecific dynamics of the fungal communities by assessing the composition and diversity of fungi among sites. We found that fully mycoheterotrophic species are more specialized in their fungal associations than chlorophyllous species, and that this specialization possibly results from the gradual loss of some fungal groups. In particular, although many fungal species were shared by different *Burmannia* species, fully MHP species typically host species-specific fungal assemblages, suggesting that they have a preference for the selected fungi. Although no apparent cophylogenetic relationship was detected between fungi and plants, we observe that evolutionarily closely related plants tend to have a greater proportion of shared or closely related fungal partners. Our findings suggest a host preference and specialization toward fungal assemblages in *Burmannia*, improving understanding of interactions between MHPs and fungi.

## Introduction

Arbuscular mycorrhizae represent a symbiotic association between land plants and arbuscular mycorrhizal fungi (AMF). AMF play a critical role in enabling plant adaptation to changing environmental conditions, and can be a driver of plant diversity [1]. The mycorrhizal symbiosis between plants and AMF is usually mutualistic, with both partners exchanging nutrients to support their respective growth: plants provide photosynthetic carbohydrates and lipids to the fungi in return for soil nutrients. In contrast, mycoheterotrophic plants (MHPs) obtain carbon and possibly also soil minerals from fungi [2, 3]. Most of these plants have lost chlorophyll and hence cannot perform photosynthesis, instead their life cycle is completely dependent on the nutrient supply from mycorrhizal fungi [2–5]. Mycoheterotrophy originated more than 40 times in the plant tree of life, with ca. 580 species from 17 families found to be putative fully mycoheterotrophic [6, 7]; among these 17 families, at least 9 have mycoheterotrophic members that are associated with AMF [6]. There are furthermore many plants that are partially mycoheterotrophic, such as *Burmannia coelestis* [8], that retain photosynthetic ability whilst also obtaining additional carbon and nutrients from AMF [9].

Previous studies of arbuscular mycorrhizal symbioses have revealed that autotrophic plant hosts and mycorrhizal fungi preferentially allocate resources to more beneficial partners, with the implication that bidirectional “partner selection” occurs in the mycorrhizal mutualism (reviewed in [10]). Investigation of the strength and importance of partner selection between MHPs and AMF under natural settings represents an invaluable contribution to studies of mycorrhizal ecology [10]. MHPs are either specialized on single fungal families, genera, or—in extreme cases—species [11], and are generally more specific to AMF than that of autotrophic plants [2, 11–15], although the degree of specificity varies by species and across families, some MHPs such as *Thismia* exhibit extremely high fungal specificity [14, 16], whereas other taxa interact simultaneously with a wide range of AMF lineages [17, 18]. These studies suggest that different MHP groups likely have divergent interaction patterns toward mycorrhizal fungi. It is speculated that the specificity of MHPs toward fungi may either be: (a) because they select for fungi more stringently, since that MHPs tend to associate with specialized and closely related fungi, providing evidence of network and/or phylogenetic constraints upon the emergence of mycoheterotrophic cheaters and their fungal partners [15] or (b) because most fungi have evolved defense mechanisms against cheating [2]. This raises many intriguing questions: for example, do independent MHP lineages select for the same fungi, implying that these fungi have a trait that makes them prone to cheating? Do MHPs recruit fungal partners locally? The specificity of mycoheterotrophs toward arbuscular fungi is furthermore expected to lead to a coevolutionary relationship, in which the evolution of plants tracks the phylogenies of their fungal partners [19]. The study of the evolutionary relationships of closely related MHPs and their fungal partners is therefore of considerable interest to botanists and evolutionary biologists. Since MHPs are usually small and ephemeral, and are often rare, restricted to well-preserved forests [4, 20], previous studies have mostly been based on only one or a few MHP species, often with comparisons between phylogenetically distant plant lineages [14, 21]: few studies have been conducted in a rigid phylogenetic framework involving species with contrasting trophic modes within a genus [15]. We aim to answer three questions: (a) What are the symbiotic associations among *Burmannia* species (Burmanniaceae) and their fungal partners? (b) How have patterns of mycorrhizal symbiosis changed during the transition from autotrophy to partial and full mycoheterotrophy? (c) To what degree do these patterns vary by lineage, species, and/or population?

*Burmannia s.l*. contains both fully mycoheterotrophic and autotrophic species [4, 22], and there is evidence that mycoheterotrophy evolved independently several times within ancestrally chlorophyllous clades [23, 24]. The genus therefore provides a rare opportunity and excellent model system for understanding the interaction between MHPs and AMF. In this study, we aim to address the gap in our understanding of the association, using high-throughput DNA sequencing methods to identify mycorrhizal fungi and test for shifts in AMF community structure across populations and species. We test the composition and diversity of AMF communities among sites to characterize fungal community dynamics within and between *Burmannia* species. By reconstructing plant and fungal phylogenies, we aim to determine possible coevolutionary relationships.

## Materials and methods

### Plant sampling

Root samples of ten *Burmannia* species were obtained from China and Malaysia, including: six achlorophyllous species (*Burmannia championii*, *B. cryptopetala*, *B. itoana*, *B. nepalensis*, *B. oblonga*, and *B. wallichii*), representing three independent origins of full mycoheterotrophy [23], two that are chlorophyllous but with vegetative reductions and hence are putatively partially mycoheterotrophic (*B. coelestis* [8] and *B. filamentosa*), and two robust chlorophyllous species (*B. disticha* and *B. longifolia*). Sampling in the study has been limited by the fact that most of *Burmannia* species are extremely rare and difficult to find: more than one third of the *ca* 60 species in the genus have been described based on only one or two collections [4, 25], and most MHPs complete their life circle within 1 month (pers. obs.). In this study, the two chlorophyllous robust species have well-developed leaves and roots and hence are categorized as autotrophs. Three species (*B. itoana*, *B. nepalensis*, and *B. wallichii*) were sampled from four populations, respectively, *B. coelestis* and *B. filamentosa* were each sampled from two populations, and samples of *B. disticha* were collected from three populations (Table S1). Samples for *B. longifolia*, *B. championii*, *B. cryptopetala*, and *B. oblonga* were only collected from one population each. All root samples were collected from flowering individuals with at least two replicates for all populations (Table S1). For each specimen, the whole root system was removed, rinsed, snap frozen using liquid nitrogen, and then stored at −80 °C for subsequent DNA extraction.

### Fungal DNA extraction, 18s rRNA gene amplification, and high-throughput sequencing

Total DNA from roots of each sample were extracted using the DNeasy Plant Mini Kit (Qiagen, Hilden, Germany). For full and partial MHPs, total DNA was extracted from the entire root system. Because mycorrhizal formation by AMF usually requires young roots for chlorophyllous plant species [26] (see also: [27]), young roots (c. 2 cm of root tips) were used to extract total DNA for *B. disticha* and *B. longifolia*, both of which have well-developed perennial roots. *Burmannia* species have been widely reported to associate with Glomeromycotina [13, 17, 28], and therefore the polymerase chain reaction (PCR) amplification was constructed with the primer set AMV4.5NF (5′-AAGCTCGTAGTTGAATTTCG-3′)/AMDGR (5′-CCCAACTATCCCTATTAATCAT-3′) with the barcode. This primer set is designed to discriminate AMF species [29] and has been widely used in various studies on AMF communities [30–33]. All PCR reactions were carried out in 30 μL reactions with 15 μL of Phusion^®^ High-Fidelity PCR Master Mix (New England Biolabs, Ipswich, MA, USA), 0.2 μM of forward and reverse primers, and about 10 ng template DNA. Thermal cycling consisted of initial denaturation at 98 °C for 1 min, followed by 30 cycles of denaturation at 98 °C for 10 s, annealing at 50 °C for 30 s, and elongation at 72 °C for 30 s, and finally at 72 °C for 5 min. Amplicons were extracted from 2% agarose gels and purified using the AxyPrep DNA Gel Extraction Kit (Axygen Biosciences, Union City, CA, USA) according to the manufacturer’s instructions and quantified using ABI StepOnePlus Real-Time PCR System (Life Technologies, Foster City, USA). Amplicon sequencing was performed using the Illumina HiSeq2500 PE250 platform.

Raw reads generated by sequencing were cleaned by removing barcodes and primer sets, and then assembled using FLASH [34] to obtain raw tags. At least 100,000 raw tags were obtained from each sample. We used the QIIME pipeline [35] and UCHIME program [36] to further filter the raw data and remove chimeric DNA sequences. The effective tags (sequences) were then clustered into operational taxonomic units (OTUs) at 97% similarity [37] using the UPARSE pipeline [38], and the most abundant sequence in each OTU cluster was selected as the representative sequence for further analysis. Unique tags that could not be assigned to any OTU cluster were removed from subsequent analyses. Taxonomic annotation of OTUs was performed using Blast search in QIIME program based on the SILVA database [39]. Only OTUs belonging to the Glomeromycotina were retained in downstream analyses.

### Diversity analysis of fungal communities

Because each sample contained a different number of reads, the abundance of OTUs of all samples were standardized by selecting the sample with the fewest reads. To assess differences among MHPs with regard to AMF specificity, we calculated abundance-based coverage estimator (ACE) and Shannon indices using QIIME: the ACE index is a measure of the number of OTUs in a fungal community, whereas the Shannon index characterizes species diversity in a community based on both abundance and evenness of the species present. Both indices are widely used to measure the microbial species α-diversity within communities, with higher values indicating higher fungal α-diversity.

To investigate the fungal diversity among different samples/groups (β-diversity), we firstly constructed an unweighted UniFrac distance matrix (UDM) [40, 41] based on the phylogenetic information of OTUs using QIIME, and then obtained a weighted UniFrac distance matrix (WUDM) using OTU abundance [42]. The two matrices were composed of UniFrac scores of pairwise samples that reflect community-wide dissimilarity, with similar fungal communities of any pair of samples having a low dissimilarity score. UniFrac measures the sequence difference of microbial communities between two samples, measured as the fraction of branch length in a phylogenetic tree [41]. UniFrac distance is the fraction of the phylogenetic tree not shared between two samples, with larger values indicating greater microbial phylogenetic difference between two samples, which may indicate greater heterogeneity among samples and presumably reflect distinct adaptation of hosts to one environment.

Based on UDM and WUDM, two-dimensional clustering/ordination and hierarchical clustering were undertaken to investigate the patterns of fungal community structure in plant samples and groups. Principal component analysis (PCoA) was performed using the packages WGCNA and stats in R. Nonmetric multidimensional scaling (NMDS) analysis was performed using the R/vegan package. Both methods are widely applied in the comparative analysis of microbial communities. In this study, PCoA and NMDS were employed to reveal relationships among samples and/or groups, with similar samples clustered together or closely positioned in the plot. In order to reveal the more specialized interactions between MHPs and AMF, we also performed a hierarchical clustering analysis of fungal communities using the unweighted pair-group method with arithmetic mean (UPGMA) in QIIME program based on both UDM and WUDM.

### Statistical analysis

Mann–Whitney *U* tests were employed to assess the significance (*p* < 0.05) of the ACE and Shannon index values between different groups using the function wilcox.test in R package status. The PERMANOVA [43] test was used to determine the significance of β-diversity between groups. We performed PERMANOVA test using the function Adonis in R package vegan.

### Plant phylogenetic analysis

18s rRNA and mitochondrial matR sequences were used in the phylogenetic analysis of Burmanniaceae. Sequences used for phylogenetic analysis were amplified from the same samples used in fungal DNA extraction, following methods described in Merckx et al. [24] and Mennes et al. [44]. *Aletris lutea* (Nartheciaceae) was chosen as the outgroup, with 18s rRNA and matR sequences downloaded from GenBank.

We independently aligned the 18s and matR sequences using MAFFT [45] plug-in in Geneious 11 [46] followed by manual inspection and adjustment. We concatenated the two alignments into a single matrix.

The phylogeny was generated using maximum likelihood (ML) methods on the IQ-TREE [47] web server. The combined matrix was partitioned by DNA region identity. DNA best-fit substitution models for each partition were selected using the ModelFinder plug-in in IQ-TREE, with TIM3e + R2 and JC + G4 identified for 18s and matR, respectively. We conducted an ultrafast bootstrap analysis with 1000 replicates.

18s rRNA sequences obtained in this study were used for the fungal phylogenetic analysis. Multiple sequence alignments were performed using M-coffee [48]. We used the ML method to construct phylogenetic trees by using PhyML 3.1 [49] with SPRs algorithms and four categories of gamma distributed substitution rates. Nonparametric bootstrap analysis was performed with 1000 replicates to estimate branch support.

### Cophylogenetic analysis

We removed outgroup taxa prior to the cophylogenetic analysis, and calculated phylogenetic distances (PDs) between plants and between fungal partners, respectively, using K2P methods implemented in MEGA [50] with transitions + transversions substitution. Fungal OTUs with relative abundance (RA) > 1% in each *Burmannia* species were used to construct the matrix of fungal PDs. We analyzed the cophylogenetic relationships between plants and their fungal partners using distance-based methods employed in ParaFit function [51] in the R package APE. ParaFit tests the hypothesis of coevolution between a clade of hosts and a clade of parasites by assessing whether parasites are randomly associated with their hosts [51]. To assess the associations between phylogenetic matrices of plants and fungal partners, we ran ParaFit for 999 permutations with lingoes correction for negative eigenvalues, and tested the significance of individual host–parasite links using the statistics ParaFitLink1 and ParaFitLink2.

To assess the effect of phylogenetic relatedness of plants on their fungal communities, we computed the Mantel test correlation between the PD matrix of plants and fungal UDM and WUDM, respectively, using Mantel function implemented in R package Ecodist [52] with 10,000 permutations.

## Results

### Fungal OTUs

**Fig. 1:**
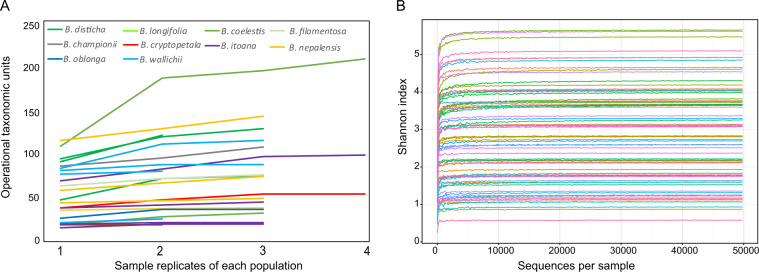
Accumulation curves for each sampled population of *Burmannia*. **A** OTU accumulation for number of sample replicates in each population of *Burmannia* species. **B** Shannon rarefaction curves for samples of *Burmannia* species.

In total, 67 DNA samples from plant roots were amplified and sequenced. Effective tags generated by high-throughput sequencing were aggregated at 97% sequence similarity, yielding 7473 fungal OTUs; among these, 5807 were assigned to the Glomeromycotina (Mucoromycota). After removing singletons, 5444 OTUs were retained, among which the most prevalent family was Glomeraceae, followed by Acaulosporaceae and Gigasporaceae (Tables S2 and S3). In addition, there are five and one single OTUs assigned to Diversisporaceae and Ambisporaceae, respectively; the OTUs from these two families were of low abundance and were much rarer than other OTUs among the plants examined in this study. Although hundreds of fungal OTUs were detected in many *Burmannia* populations, most of them are of extremely low abundance with RA < 0.01% (Tables S1 and S3) and were detected only in few samples, and they are likely opportunistic fungi that are specific to a certain region within the range of *Burmannia* and hence were removed from downstream analyses. Finally, 496 OTUs were retained in the following analysis. The most abundant OTUs were OTU0001, OTU0002, OTU0003, and OTU0004 in the genus *Glomus*, with RA exceeding 10%, and were also the most prevalent OTUs detected in most species examined. We assessed whether sufficient sampling and sequencing had been achieved by constructing species accumulation curves for each population of each plant species and Shannon rarefaction curves for all samples. Results showed that both reached a saturation plateau, indicating that our sampling and sequencing depths were sufficient to capture most of the AMF OTUs (Fig. 1A, B).

### Analysis of fungal α-diversity

**Fig. 2:**
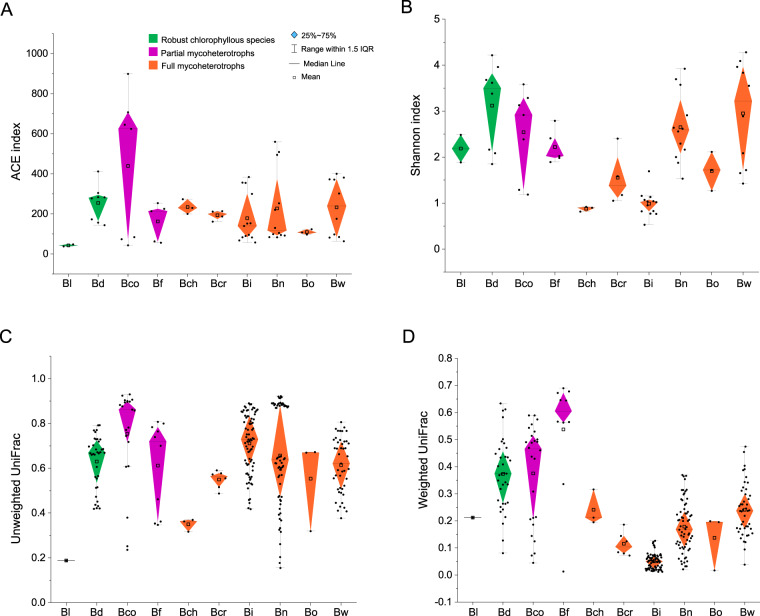
Within-species α- and β-diversity of sampled *Burmannia* species. **A** ACE index. **B** Shannon index. Black dots represent the values of ACE index (**A**) and Shannon index (**B**) for each sample, respectively. **C** Unweighted UniFrac distance distribution within species. **D** Weighted UniFrac distance distribution within species. Black dots represent within-species UniFrac distances between samples. Bl *B. longifolia*, Bd *B. disticha*, Bco *B. coelestis*, Bf *B. filamentosa*, Bch *B. championii,* Bcr *B. cryptopetala,* Bi *B. itoana*, Bn *B. nepalensis*, Bo *B. oblonga*, Bw *B. wallichii*.

*Burmannia* species varied in their fungal α-diversity (quantified with ACE and Shannon diversity indices within each individual). For autotrophs, although *B. disticha* had higher fungal α-diversity than most other *Burmannia* species examined in this study, the other autotrophic species, *B. longifolia*, which usually grows among mosses and is sometimes epiphytic, had a much lower fungal diversity than most congeners, including full mycoheterotrophs (Fig. 2A, B, Table S4). One of the two partial mycoheterotrophs, *B*. *coelestis*, had a high fungal diversity with the highest average ACE indices (Fig. 2A), while the other one, *B*. *filamentosa*, had lower fungal diversity. Some achlorophyllous MHPs, such as *B. itoana* and *B. oblonga*, had a low fungal diversity, whereas others, such as *B. wallichii* and *B. nepalensis*, had a higher fungal diversity similar to the autotrophic *B. disticha* (Fig. 2B). There was no significant difference between different trophic groups (*p* > 0.05, Mann–Whitney *U* test).

If the fungal diversity in MHPs was governed by the local environment, co-occurring populations (intermingled in the same site) should have similar fungal α-diversities, which means that they have similar fungal species and/or similar numbers of fungi. To test whether the fungal diversity in MHPs was governed by the local environment, we therefore compared fungal α-diversities for co-occurring populations of *B. itoana* and *B. cryptopetala* from Guanyin Mountain, and similarly for intermingled populations of *B. itoana* and *B. nepalensis* from Chenhedong (Table 1). We also calculated coefficients of variation (CVs, the ratio of the standard deviation to the mean) for ACE and Shannon indices to test the degree of variation of α-diversity within each population. All four populations showed low diversity with within-population ACE indices (CV < 0.15), while only *B. nepalensis* 4 showed low variability with within-population Shannon indices (CV = 0.04). For the ACE index, all interspecific comparisons of differences are statistically significant. For the Shannon index, the interspecific comparison between *B. itoana* 4 and *B. nepalensis* 4 was statistically significant, although the comparison between *B. cryptopetala* and *B. itoana* 1 was not.

**Table 1:** Tests for the difference of α-diversity between populations of coexisting *Burmannia* species.

		Coefficient of variation (CV)		Mann–Whitney *U* test	
Comparisons	Population	ACE index	Shannon index	ACE index	Shannon index
*B. cryptopetala* vs *B. itoana* 1	*B. cryptopetala*	0.11	0.34	*p* < 0.05	*p* > 0.05
	*B. itoana* 1	0.04	0.16		
*B. itoana* 4 vs *B. nepalensis* 4	*B. itoana* 4	0.09	0.33	*p* < 0.05	*p* < 0.05
	*B. nepalensis* 4	0.05	0.04		

### Fungal composition among species and populations

Most OTUs were shared by more than one plant species, although their abundance varies greatly between populations and species. Although several plant species shared some highly abundant OTUs, each of them typically has a different fungal OTU that is most abundant (Tables S2 and S3).

To examine whether the fungal composition varied within/between populations (β-diversity), we calculated UniFrac distances among samples (Table S5). Results showed that β-diversity for unweighted UniFrac distances varied greatly among plant species: *B. longifolia* had the lowest and *B. coelestis* had the highest within-species β-diversity (Fig. 2C). The between-population fungal PD was no more significantly different among populations within plant species than among populations across species; however, most samples could have low fungal PDs with inter- or intraspecies populations (Fig. S1), for example, samples of *B. disticha* showed low UniFrac distances with several species, such as *B. wallichii*, *B. cryptopetala*, and *B. championii* (Fig. S1), possibly because they largely share fungal OTUs.

Unweighted UniFrac does not account for the abundance of sequences in different samples; however, since the abundance of different fungi can be critical for describing community changes, we therefore calculated weighted UniFrac (W-UniFrac) distances by accounting for the abundance of fungal OTUs (Table S6). Chlorophyllous species generally had much higher within-species W-UniFrac distances than achlorophyllous species (Fig. 2D): the chlorophyllous species *B. filamentosa* had the highest distances, whereas the achlorophyllous species *B. itoana* had the lowest. Several mycoheterotrophic species, such as *B. itoana*, *B. wallichii*, and *B. nepalensis*, had significantly low within-species W-UniFrac distances but higher interspecies distances (Fig. S2).

We used PERMANOVA test to examine whether the fungal communities in different *Burmannia* populations and/or species were significantly different from each other. Based on UniFrac and W-UniFrac distances, the significance of the between-species and within-species variation were tested (Tables 2 and 3). Populations with at least three sampling replicates were used for the within-species comparisons. In most cases the tests between different samples within one species are not statistically significant, while most between species are statistically significant, demonstrating that the fungal composition is more distinct between species than within species. Co-occurring populations of *B. itoana* and *B. cryptopetala* and those of *B. itoana* and *B. nepalensis* showed significant between-species variation but within-species variation was not significant (Table 2).

**Table 2:** PERMANOVA tests for the degree of compositional difference between populations.

	Unweighted UniFrac		Weighted UniFrac	
Comparisons	*R*^2^	*p* value	*R*^2^	*p* value
*B. coelestis* MA vs *B. coelestis* HN	0.70	0.03	0.51	0.03
*B. itoana* 1 vs *B. itoana* 2	0.79	0.10	0.78	0.10
*B. itoana* 2 vs *B. itoana* 3	0.20	1.00	0.80	0.10
*B. itoana* 3 vs *B. itoana* 4	0.63	0.04	0.61	0.10
*B. nepalensis* 1 vs *B. nepalensis* 2	0.30	0.60	0.75	0.10
*B. nepalensis* 2 vs *B. nepalensis* 3	0.51	0.10	0.82	0.10
*B. nepalensis* 3 vs *B. nepalensis* 4	0.53	0.10	0.50	0.20
*B. wallichii* B vs *B. wallichii* H1	0.30	0.10	0.04	0.80
*B. disticha* 2 vs *B. disticha* 3	0.67	0.10	0.33	0.20
*B. cryptopetala* vs *B*. *itoana* 1^a^	0.52	0.06	0.96	0.03
*B. itoana* 4 vs *B. nepalensis* 4^a^	0.05	1.00	1.00	0.03

^a^Co-occurring populations from different *Burmannia* species.

**Table 3:**
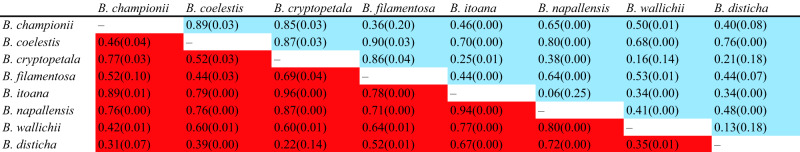
PERMANOVA tests for the degree of fungal compositional difference between pairs of *Burmannia* species.

*R*^2^ (*p* value) based on unweighted UniFrac are in blue and those based on weighted UniFrac are in grey.

### Patterns of fungal community structure

**Fig. 3:**
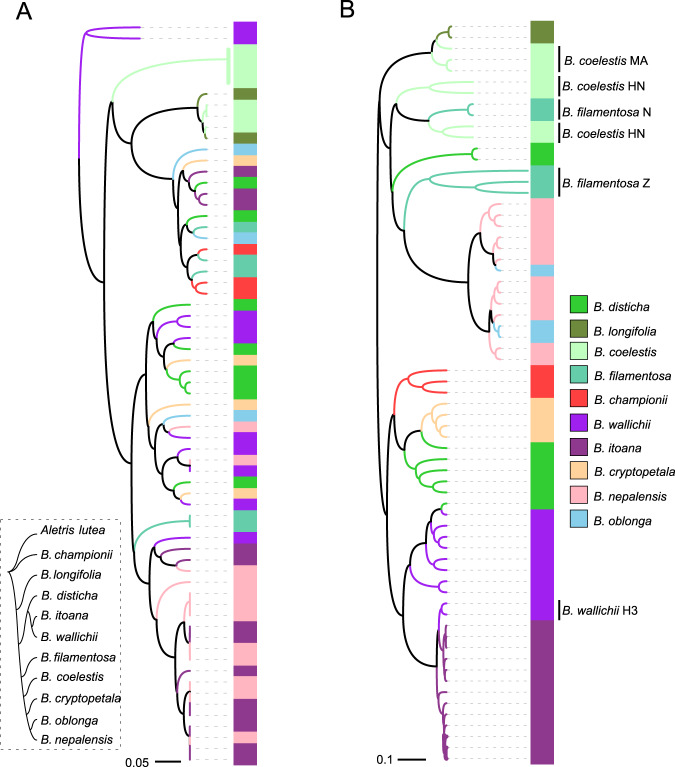
UPGMA analysis for fungal communities in *Burmannia* populations. **A** unweighted UniFrac distances and **B** weighted UniFrac distances. Intraspecies populations that fall into different clusters are indicated. Leaves represent plant samples. A phylogenetic tree of *Burmannia* constructed in this study is shown at the lower left.

**Fig. 4:**
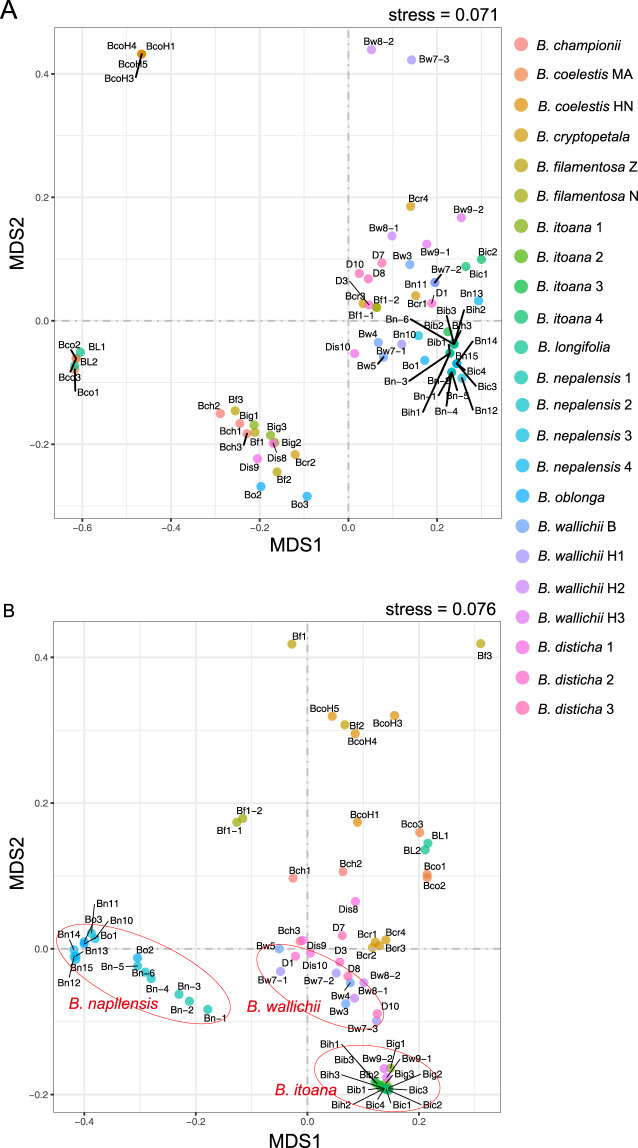
Nonmetric multidimensional scaling analysis for fungal communities in *Burmannia* populations. **A** unweighted UniFrac distances and **B** weighted UniFrac distances, where color denotes the population of *Burmannia* species.

In order to better visualize the overall patterns of diversity, we used UniFrac and W-UniFrac distances to perform clustering analysis. The UPGMA analysis based on UniFrac and W-UniFrac data resulted in different clustering patterns. In the results derived from UniFrac, samples from one species were discrete (Fig. 3A), while in the results derived from W-UniFrac, samples from several species such as *B. itoana* and *B. nepalensis* were species specifically clustered (Fig. 3b). Most samples of *B. wallichii* were clustered together, forming a group closely neighboring *B. itoana*, while two *B. wallichii* samples were surprisingly aggregated within the *B. itoana* cluster; similarly, all samples of *B. oblonga* fell into the *B. nepalensis* cluster. The fungal composition from samples of the autotrophic species *B. disticha* was clustered into different groups. For *B. coelestis* and *B. filamentosa*, samples were clustered into different groups, with each cluster comprising samples from only one population (Fig. 3B). The results of the NMDS (Fig. 4) and PCoA (Fig. S3) analyses were similar to the UPGMA results.

### Phylogenetic analysis

**Fig. 5:**
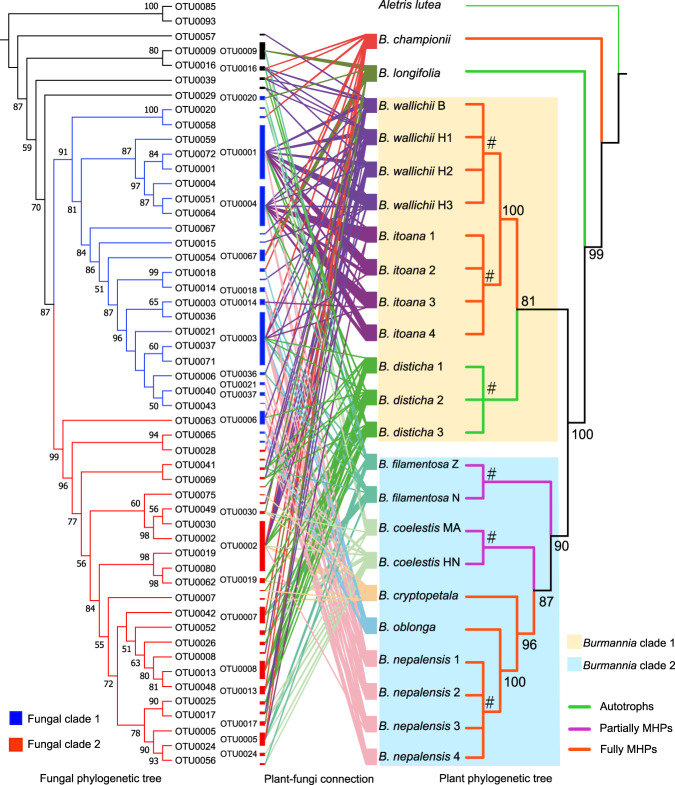
Plant–fungi connection network based on phylogenetic relationships. Ultrametric trees for sampled *Burmannia* species (right) and associated AMF (left) were generated based on ML tree, respectively. Numbers on major branches indicate bootstrap support (>50%). *Aletris lutea* (Nartheciaceae) was used as the outgroup for the plant phylogenetic tree, and Otu0085 and Otu0093 (Acaulosporaceae) were used as the outgroup for fungal phylogenetic tree. Lines in the plant–fungi connection network indicate the detection of fungal OTUs in each *Burmannia* species, and the line width is proportional to the abundance of one fungal OTU in that plant species. The length of fungal OTU bar in the plant–fungi connection network represents the number of connections for that OTU, and OTUs with high abundance (overall relative abundance > 0.5%) are presented. Symbol “#’” marks different populations for that *Burmannia* species. MHPs mycoheterotrophic plants.

To examine whether there are coordinate phylogenetic relationships between plant and fungal phylogenies, we constructed phylogenetic trees for plants and fungi, respectively (Figs. S4 and S5). Results showed that the *Burmannia* species studied here were well resolved, with *B. championii* and *B. longifolia* forming the first and second diverging lineages, respectively. The remaining species formed two distinct clades comprising MHPs and autotrophs: clade 1, with *B. wallichii*, *B. itoana*, and *B. disticha*, and clade 2, with *B. cryptopetala*, *B. nepalensis*, *B. oblonga*, *B. filamentosa*, and *B. coelestis* (Fig. S4). In the fungal phylogeny, five OTUs formed an early divergent grade, and all others forming two well supported clades (Fig. S5). We found no significant cophylogenetic signal between the two trees (ParaFitGlobal = 0.0017, *p* value = 0.655): each of the two main plant clades associate with members of both of the major fungal clades, resulting in reticulate connections among plants and fungi (Fig. 5). As the cophylogeny may occur only between plants and their dominant fungal partners, cophylogenetic signals between plants and their fungal OTUs with RA > 10% (see Table S7 for dominant fungal OTUs) were also examined. However, no significant cophylogenetic signal was detected (ParaFitGlobal = 0.00045, *p* value = 0.232), either. The Mantel test, however, showed that closely related plant species tend to have more closely related fungal communities (Mantel test: *p* < 0.05, Table 4): *B. nepalensis* and *B. oblonga*, for example, are closely related in the phylogenetic tree and share most of their fungal partners (Figs. 3b and 5), as do *B. itoana* and *B. wallichii*. Notably, the Mantel test showed a much more significant correlation between plant species and fungal communities based on WUDM (*R* = 0.66, *p* < 0.01) than UDM (*R* = 0.13, *p* < 0.05).

**Table 4:** Mantel test between the phylogenetic distances of plant species and the pairwise distances of their fungal communities.

Fungal distance matrix	Mantel *R*	*p* value	Permutations
UniFrac distance	0.13	0.02	10,000
Weighted UniFrac distance	0.66	0.00	10,000

## Discussion

Our survey of the diversity of fungal symbionts associated with the roots of autotrophic, partially mycoheterotrophic and fully MHPs revealed that *Burmannia* species are generally able to host numerous fungal partners simultaneously and thus reveal less specificity toward AMF than *Thismia* species (Thismiaceae) [14, 16]. Although some mycoheterotrophic species have high fungal α-diversity, they all had much lower β-diversity when compared with autotrophic congeners, supporting the hypothesis that mycoheterotrophs are more specialized with regard to fungal partners than related autotrophs [14, 15]. Interestingly, the autotrophic species *B. disticha* had the highest α-diversity of fungal partners, with most fungal partners shared with other mycoheterotrophic lineages. We furthermore found no significant coordinate phylogenetic signal between plant and fungal phylogenetic trees, suggesting the absence of phylogenetic constraints between *Burmannia* species and their fungal partners.

### Specialization toward Glomeraceae may result from the gradual loss of fungal partners in *Burmannia*

The great majority of Glomeromycotina sequences obtained from *Burmannia* species in this study are assigned to Glomeraceae, corroborating previous studies [13, 28]. Although non-Glomeraceae OTUs including taxa belonging to two Glomeromycete families (Gigasporaceae and Acaulosporaceae) were also retrieved, these were abundant in the autotrophic species *B. disticha*, less common in the partially mycoheterotrophic species *B. coelestis* and *B. filamentosa*, and absent from most of the fully mycoheterotrophic *Burmannia* species. These findings suggest that *Burmannia* species gradually abandoned these non-Glomeraceae fungi according to their trophic modes during the evolution of full mycoheterotrophy, supporting the hypothesis that full or partial loss of photosynthesis selects for different mycorrhizal communities [7]. As with Glomeraceae, fungi in Gigasporaceae and Acaulosporaceae can also form mycorrhizae with most plants: they are slow to colonize host plants [53] and are generally less efficient than Glomeraceae at increasing plant biomass [54], although there is considerable variability between fungi in their effects on different host plants [54]. The precise mechanisms underlying the exclusion of these fungal taxa in the mycorrhizae of MHPs, however, need to be further investigated.

### Mycoheterotrophic *Burmannia* species tend to associate with specialized fungal assemblages

Recent studies have revealed that fungal partners can be shared among different mycoheterotrophs [55, 56]. Our results are consistent with this, indicating that most fungal OTUs are generally shared by more than two mycoheterotrophic *Burmannia* species (Table S2): for example, Otu0001 is mainly shared by *B*. *itoana*, *B*. *nepalensis*, and *B*. *wallichii*, and Otu0002 is mainly shared by *B*. *disticha*, *B*. *championii*, and *B*. *cryptopetala*. The pattern in which MHPs associate with close relatives of fungal partners remains a mystery, however. Our results indicate that the abundance of different fungal OTUs, besides the composition of OTUs, are likely critical for describing community changes in *Burmannia*, suggesting that *Burmannia* plants tend to have species-specific preference for the selected fungi. This trend was much more marked in full MHPs: all samples of *B. itoana* and *B. nepalensis*, the two species representing independent lineages of MHPs in the genus, for example, formed species-specific clusters in the UPGMA tree, and most *B. wallichii* samples similarly clustered together. These results suggest that fully MHPs are not associated with a random assortment of fungi, but are instead associated with a specific assemblage of fungi, even though the number or abundance of fungal partners might vary in different within-species populations or in different individuals within one population. Populations of the fully MHP species studied here are geographically distant from each other and occur at different elevations and with very distinct populations of sympatric plant species. MHPs such as *B. wallichii* may be mostly self-pollinated, resulting in high degree of intraspecific differentiation [57]. We furthermore found that different MHPs intimately co-occurring in the same locality (for example, *B. itoana* 4 and *B. nepalensis* 4) had the same accompanying plants but significantly distinct fungal associates (Table 2), suggesting that the specialization toward fungal assemblage in MHPs are unlikely to be caused by the preference toward fungi of associated autotrophic plants. Although various environmental factors—such as temperature, soil organic matter, habitat types, or most probably neighboring autotrophs—can influence the diversity of fungal partners [58–62], it appears unlikely that environmental factors are the exclusive mechanisms underlying the specialized assemblages of fungal partners in MHPs. Distinct fungal compositions between MHP species are therefore more likely defined by interspecific genetic differentiation of plants, and less by the availability of local fungal partners. Notably, although the MHPs investigated here are from different evolutionary lineages (Fig. 5), they possessed the same interaction patterns toward fungal partners: all of them showed preference to host a specific assemblage of fungi. We therefore propose that the specificity toward fungal assemblages is likely to be stable in mycoheterotrophic *Burmannia* species, regardless of location or habitat. This tendency is interestingly reduced in partially mycoheterotrophic species (*B. coelestis* and *B. filamentosa*), in which fungal communities clustered into different UPGMA groups, suggesting that there were significant intraspecific differences in their fungal assemblages. However, they still showed high fungal assemblage preference within populations. We found that populations of *B. filamentosa* shared most of their highly abundant fungal OTUs, and the difference in their fungal associations might be attributable to the influence of different plant neighbors and/or the availability of fungal partners. Populations of *B. coelestis* shared few fungal OTUs with a significant difference in abundance and thus are unlikely to be restricted to certain fungi. Considering that the two populations of *B. coelestis* sampled were geographically distant and occupy different climatic zones, the fungal assemblages were more likely shaped by differentiated habitats, in which fungal community composition is strongly influenced by the local environment [58], and that autotrophs usually exhibit less specificity toward fungal partners [14, 15].

### Symbiotic balancing between specialization toward fungal assemblage and fungal diversity

Many autotrophs have been shown to discriminate fungi at a fine scale [1, 10] with preferential allocation of resources to higher quality partners [10], while fungal partners have different capabilities to take up and supply nutrients to plants [63]. It seems that MHPs overturn this pattern of interaction by seizing nutrients from fungi that are probably most effective for nutrient uptake of plant hosts, and the specialization toward fungal assemblages may be helpful in improving nutritional efficiency for plant parasites. Indeed, intraradical (within the root) competitive interactions between AMF species could reduce overall fungal abundance in the plant host and is most intense for resources within the plant host [64], and increasing fungal diversity does not necessarily increase benefits to plants [65]. At the same time, most MHPs are highly adapted to their environment, and the specific interactions toward fungal mutualists may limit their geographical distributions [66]. Moreover, it has been reported that the abundance of fungal partners may determine the distribution of MHPs [67]. The specificity toward fungal assemblages might therefore be a balance of maximizing benefit from its fungal partners and increasing diversity of fungal partners to explore niche breadth. This could explain why some mycoheterotrophic *Burmannia* species, such as *B. itoana* and *B. nepalensis*, are widespread and often comprise large populations, whereas other mycoheterotrophic genera, such as *Thismia*, which exhibit extreme fungal specificity, usually have limited distribution ranges and have small populations [16].

One explanation for why specificity toward fungi occurred in full MHPs is that many fungi may have escaped from parasitic plant hosts [2]. Our results showed that independent fully MHP lineages tend to select for the same fungi, implying that these fungi failed to escape from certain parasitic plant hosts or they have a trait that makes them prone to cheating. However, no evidence has been provided that fungi have immunity mechanisms to resist the invasion of parasitic plants. Whether the specificity results from the escaping of fungi therefore remains to be confirmed.

The variation in fungal assemblages among MHPs may also be of ecological importance and could help to explain the co-existence of MHPs. Plants with similar niches may be under intense competition for the same resources [68]. As MHPs are completely dependent on AMF, coexisting populations may experience intense interspecies competition for nutrients if they share the same mutualistic partner for the commodities provided by that shared partner [69]. Specialization on differentiated fungal assemblages could therefore help the coexisting species explore their own ecological niche, reducing interspecies competition and improving fitness. Similarly, fungal diversity appears to be in direct proportion to the fungal overlap among MHP hosts, which is possibly a survival strategy to maximize co-occurrence and avoid competitive exclusion among MHPs [56].

### Cophylogenetic relationship between fungal partners and MHPs was not apparent

A compelling alternative explanation for why specialization occurred in the relationships of most MHPs and their fungal partners is that both engaged in a coevolutionary “arms race,” possibly leading to cocladogenesis between plants and fungi [19]. Our results revealed no apparent concordance between the phylogenies of *Burmannia* and their fungal partners (Fig. 5), and it therefore seems unlikely that plant parasites in *Burmannia* phylogenetically coevolved with their fungal partners. We nevertheless observed that evolutionarily closely related plant species—such as *B. oblonga* and *B. nepalensis*, *B. itoana* and *B. wallichii*—tended to have a greater number of shared or closely related fungal partners (Fig. 5). Moreover, the fungal communities associated with two partially mycoheterotrophic species, *B. filamentosa* and *B. coelestis*, were also closely clustered (Figs. 3 and 4), suggesting that they have a similar fungal composition. These two partial MHPs are also phylogenetically close, and survive in quite similar habitats [70]. These results, together with the fact that the robust chlorophyllous species *B. disticha*, which is mostly autotrophic, tended to share most of its fungal partners with mycoheterotrophic relatives, suggest that MHPs, while experiencing host divergence and specialization during evolution, may inherit fungal partners from their autotrophic ancestors [15]. Species sampling was constrained in the present study, and additional sampling might provide further evidence for this; if this is the case, it is quite possible that shift/divergence of fungal partners frequently occurred within each lineage leading to full mycoheterotrophs, and the shifts in fungal partners most probably occurred after the species divergence in *Burmannia*.

## Conclusion

Our analysis of autotrophic and mycoheterotrophic *Burmannia* species and their interactions with AMF illustrates a pattern of plant–fungi interaction which has never been reported before, in which mycoheterotrophic *Burmannia* species tend to associate with more specialized fungal assemblages by exhibiting high preference for selected fungi; although cophylogenetic relationship between fungal partners and MHPs was not detected, closely related plant species tend to have more similar fungal partners. Such host preference and specialization toward fungal assemblages in *Burmannia* probably reflect a symbiotic balance between specialization and diversity of fungal partners. This pattern of interaction provides a new perspective for assessing the nutritional strategies of MHPs and their interactions with fungi.

## Supplementary information

Supplementary Information

Figure S1

Figure S2

Figure S3

Figure S4

Figure S5

Table S1

Table S2

Table S3

Table S4

Table S5

Table S6

Table S7
