# Supplementary material for: Specificity of assemblage, not fungal partner species, explains mycorrhizal partnerships of mycoheterotrophic Burmannia plants
Source: ISME J. 2021 Jan 6;15(6):1614–27. doi: 10.1038/s41396-020-00874-x (PMC8163756; doi:10.1038/s41396-020-00874-x)
Supplement: Supplementary file 1 — Supplementary Information [file 41396_2020_874_MOESM1_ESM.docx]

**Supplementary Figures
Fig. S1** Unweighted UniFrac distances between samples of *Burmannia* species.

**Fig. S2** Weighted UniFrac distances between samples of *Burmannia* species.

**Fig. S3** Principal Component Analysis (PCoA) for (A) unweighted UniFrac distances and (B) weighted UniFrac distances, where color denote the population of *Burmannia* species.

**Fig. S4** Maximum likelihood tree for *Burmannia* species. 18s rRNA and mitochondrial matR sequences were used for the phylogenetic analysis. *Aletris lutea* (Nartheciaceae) was used as the out group. Numbers on branches indicate bootstrap support.

**Fig. S5** Maximum likelihood tree for fungal partners identified in *Burmannia* species examined in this study. 18s rRNA gene sequences obtained in this study were used for the phylogenetic analysis. Otu0085 and Otu0093 (Acaulosporaceae) obtained in this study were used as the out group for fungal phylogenetic tree. Numbers on branches indicate bootstrap support.

**Supplementary Tables
Table S1** Sampling location and sample replicates (individuals) for populations of *Burmannia* species.

**Table S2** Abundance of fungal OTUs in each sample from *Burmannia* species.

**Table S3** Relative abundance of fungal OTUs in each sample from *Burmannia* species.

**Table S4** Alpha diversity of fungal hosts in each *Burmannia* species.

**Table S5** UniFrac distances for fungal assemblages in *Burmannia*.

**Table S6** Weighted UniFrac distances for fungal assemblages in *Burmannia*.

**Table S7** Dominant fungal OTUs with RA >10% in each *Burmannia* sample.
