# Supplementary figures and images for: Specificity of assemblage, not fungal partner species, explains mycorrhizal partnerships of mycoheterotrophic Burmannia plants
Source: ISME J. 2021 Jan 6;15(6):1614–27. doi: 10.1038/s41396-020-00874-x (PMC8163756; doi:10.1038/s41396-020-00874-x)

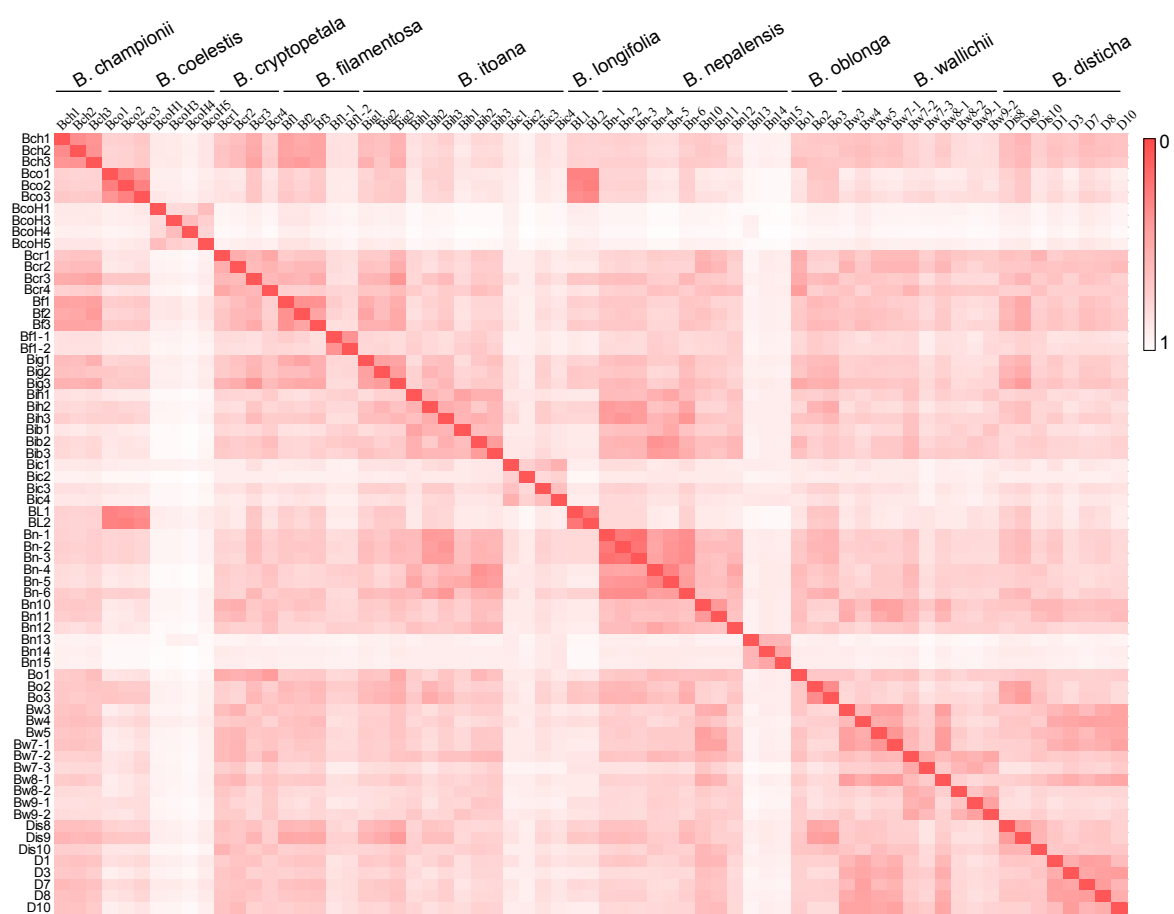

Supplement: Supplementary file 2 — Figure S1 [file 41396_2020_874_MOESM2_ESM.pdf]

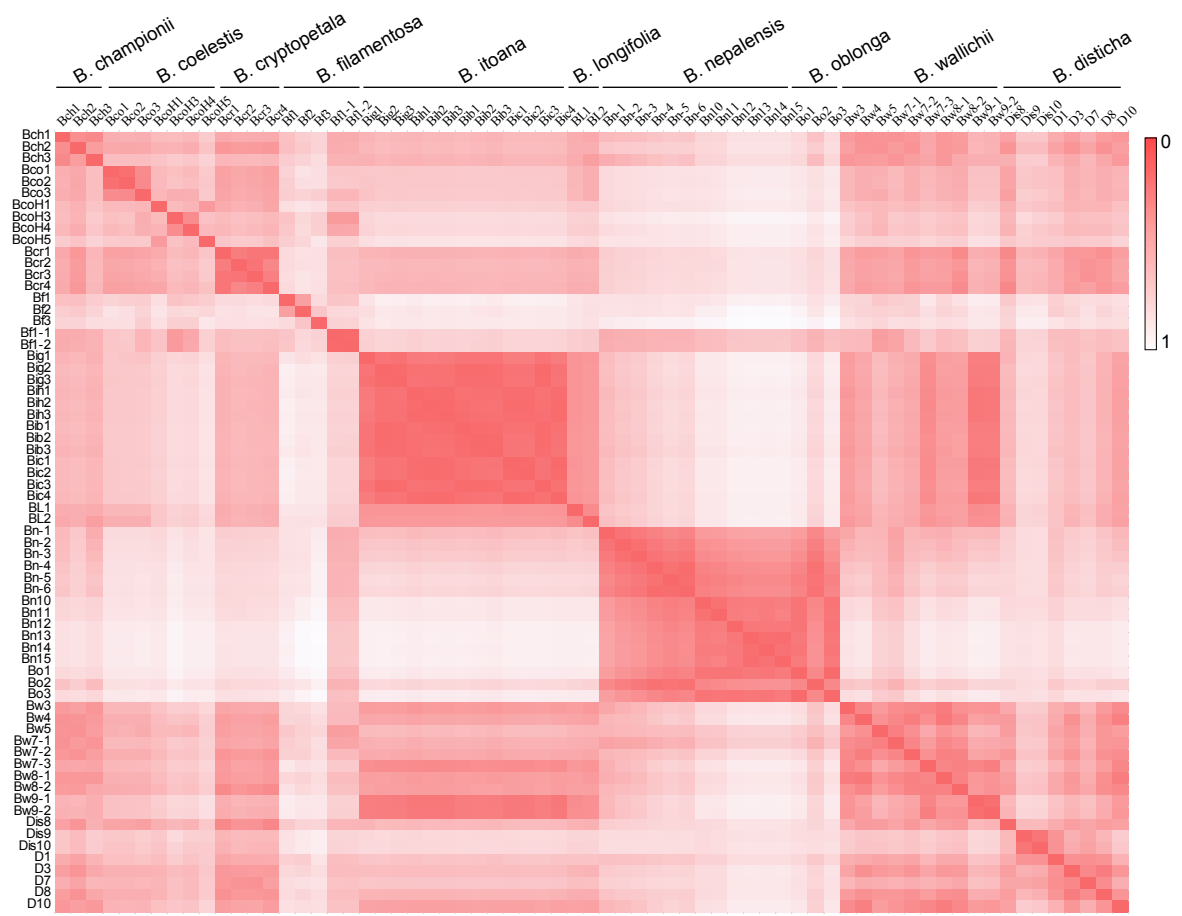

Supplement: Supplementary file 3 — Figure S2 [file 41396_2020_874_MOESM3_ESM.pdf]

B

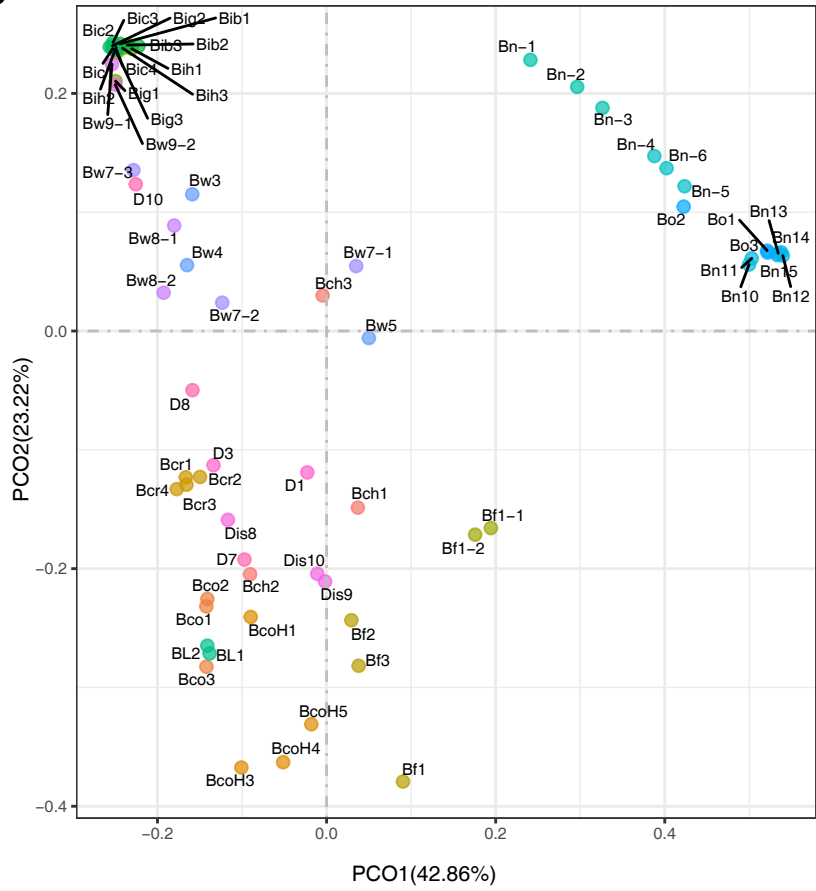

Supplement: Supplementary file 4 — Figure S3 [file 41396_2020_874_MOESM4_ESM.pdf]

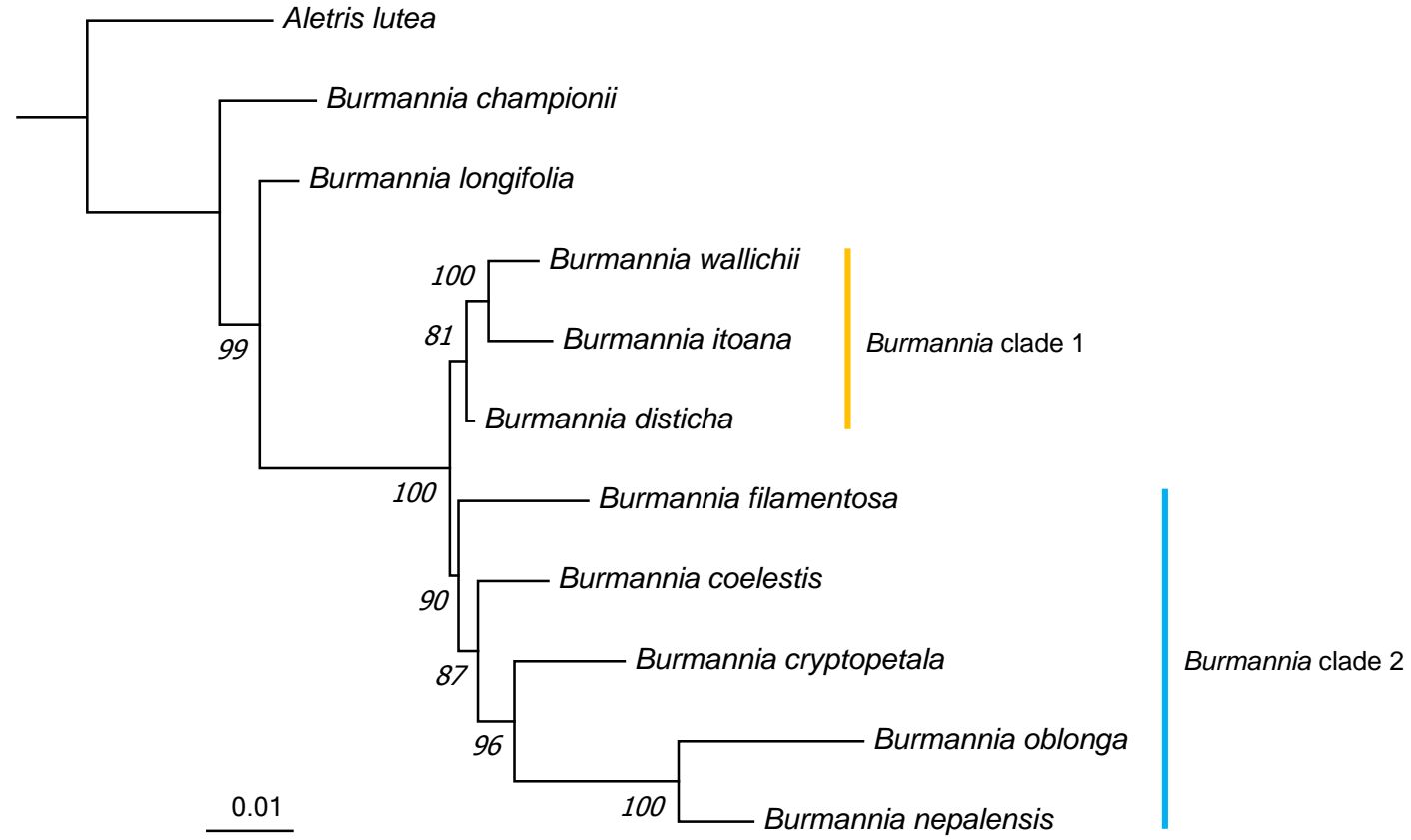

Supplement: Supplementary file 5 — Figure S4 [file 41396_2020_874_MOESM5_ESM.pdf]

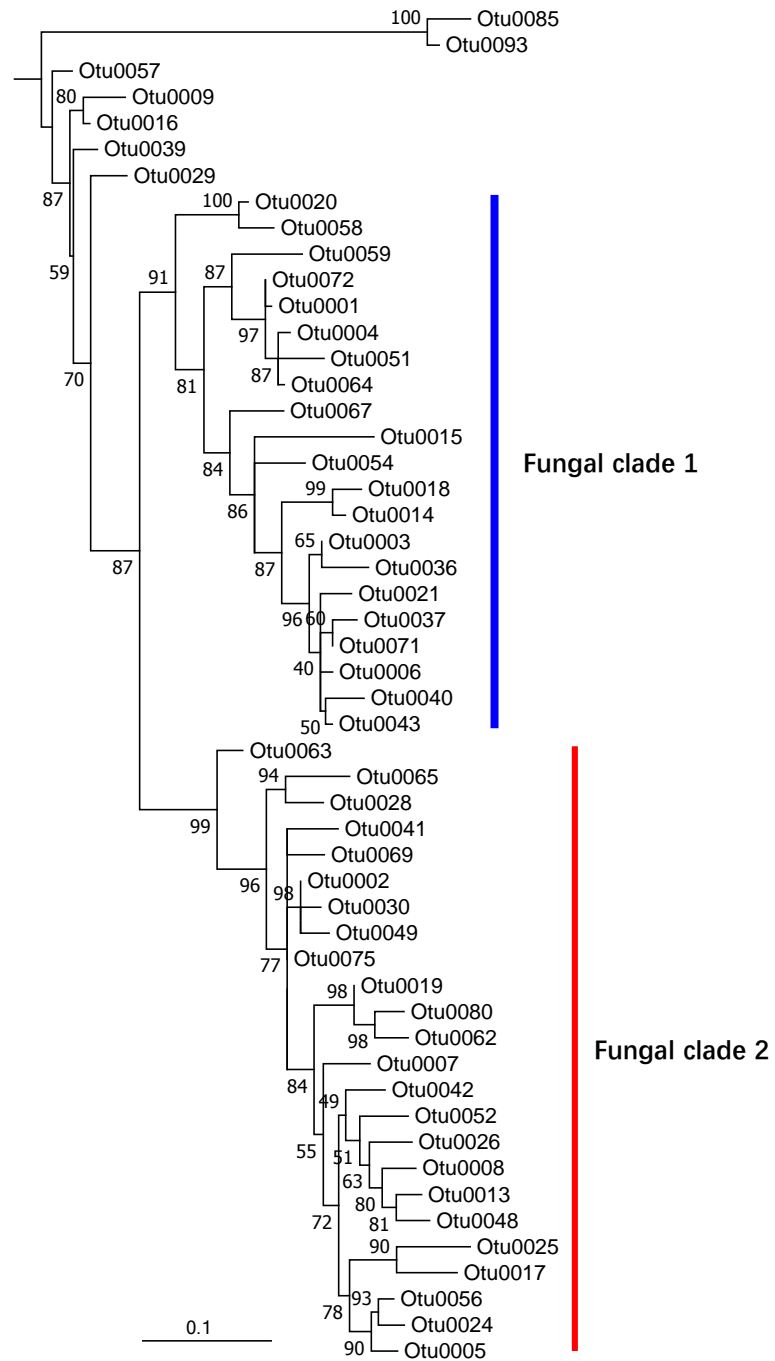

Supplement: Supplementary file 6 — Figure S5 [file 41396_2020_874_MOESM6_ESM.pdf]
